# Beyond Rifampin: Evaluating Rifapentine and Rifabutin as Alternative Treatments for Tuberculous Meningitis

**DOI:** 10.1093/infdis/jiag087

**Published:** 2026-08-03

**Authors:** Xueyi Chen, Carlos E. Ruiz-Gonzalez, Yuderleys Masias-Leon, Medha Singh, Oscar J. Nino-Meza, Charles A. Peloquin, Dmitri Artemov, Sanjay K. Jain

**Affiliations:** 1 Department of Pediatrics, Johns Hopkins University School of Medicine, Baltimore, Maryland, USA; 2 Center for Tuberculosis Research, Johns Hopkins University School of Medicine, Baltimore, Maryland, USA; 3 Department of Pediatrics, Cincinnati Children’s Hospital Medical Center, Cincinnati, Ohio, USA; 4 Cincinnati Children’s Center for Molecular Imaging and Precision Medicine, Cincinnati Children’s Hospital Medical Center, Cincinnati, Ohio, USA; 5 Infectious Disease Pharmacokinetics Laboratory, Pharmacotherapy and Translational Research, University of Florida College of Pharmacy, Gainesville, Florida, USA; 6 Department of Radiology and Radiological Science, Johns Hopkins University School of Medicine, Baltimore, Maryland, USA

**Keywords:** tuberculosis, *Mycobacterium tuberculosis*, tuberculous meningitis, rifamycins

## Abstract

**Background.:**

Tuberculous meningitis (TB meningitis), the most severe form of tuberculosis, carries high mortality and neurological sequelae, and current rifampin-based regimen are limited by poor central nervous system penetration. Alternative rifamycins like rifapentine and rifabutin, with different pharmacokinetic profiles, are effective for treating drug-susceptible pulmonary tuberculosis, with rifapentine now forming the basis of a World Health Organization-approved 4-month daily rifapentine-moxifloxacin regimen, and retrospective studies showing that rifabutin-based regimens are effective and well tolerated, however, both drugs still need dedicated evaluation in TB meningitis.

**Methods.:**

Mice were intracranially inoculated with *Mycobacterium tuberculosis* and treated with rifamycin (rifampin, rifapentine, or rifabutin)-based regimens at human equipotent doses. We assessed the bactericidal activities of the 3 regimens, rifamycin tissue concentrations, brain inflammation (brain magnetic resonance imaging, tissue immunofluorescence, cytokines), and neuronal injury.

**Results.:**

Both rifapentine- and rifabutin-containing regimens demonstrated bactericidal activity in the brain, similar to or better than the standard rifampin-containing regimen, as well as reduced neuroinflammation and brain injury.

**Conclusions.:**

Alternate rifamycins, rifapentine and rifabutin, show therapeutic activity and neuroprotective effects, supporting their evaluation in clinical trials for treating TB meningitis.

Tuberculous meningitis (TB meningitis), the most severe form of tuberculosis, is associated with high rates of mortality and neurological disability despite current treatments, and poses significant diagnostic and therapeutic challenges due to immunopathology being a key driver of central nervous system (CNS) damage [1, 2]. Current regimens, adapted from treatment for pulmonary tuberculosis, often fail to achieve adequate drug concentrations in the CNS [3], exacerbating the risks for poor clinical outcomes.

The rifamycin antibiotics—rifampin, rifapentine, and rifabutin—are the cornerstone antibiotics for tuberculosis treatments, yet their efficacy in TB meningitis remains constrained by variable blood-brain barrier (BBB) penetration [2]. The standard tuberculosis treatment utilizes rifampin, which exhibits dose-dependent efficacy but achieves subtherapeutic CNS concentrations at standard doses [4-6]. Higher doses of rifampin are being evaluated to overcome its limited CNS penetration; however, clinical trials data regarding efficacy remain conflicting [7-12]. Alternative rifamycins, such as rifapentine and rifabutin, offer differing pharmacokinetic profiles and have been utilized in regimens for pulmonary tuberculosis with success [13]. Preclinical and early clinical data have shown that rifabutin achieves adequate concentrations in the meninges, spinal cord, and brain tissue, and, unlike rifampin, does not induce hepatic metabolism of other drugs [14, 15]. Rifapentine, notable for its long half-life and potent sterilizing activity, has been combined with moxifloxacin, enabling shorter (4 months) and effective treatment for drug-susceptible pulmonary tuberculosis [16]. Taken together, these findings highlight the need to optimize rifamycins use in TB meningitis by exploring higher doses, new combinations, and regimens tailored to enhance drug delivery to the CNS.

This article provides data from a mouse model of TB meningitis [3, 5, 17] to evaluate the pharmacodynamic properties, inflammatory effects, and comparative efficacy of these rifamycins, addressing a critical knowledge gap in optimizing treatments for TB meningitis. To address clinically relevant questions, we used human-equipotent doses and, where relevant, regimens that are approved for clinical use [5, 18-21]. Rifampin (R) was evaluated at a human dose of 10 mg/kg/day combined with isoniazid (H) and pyrazinamide (Z) (HRZ), and compared with rifabutin at a human dose of 300 mg/day (HRbZ). Rifapentine (P) was evaluated in combination with isoniazid, pyrazinamide, and moxifloxacin (Mx), at a human dose of 1200 mg/day. The HPZMx regimen is recommended by the World Health Organization for pulmonary tuberculosis utilizing daily rifapentine dosing [16, 21].

## METHODS

### Study Approval

All protocols were approved by the Johns Hopkins University Biosafety, Radiation Safety, Animal Care and Use Committees (MO19M382).

### Animal Studies

Female C3HeB/FeJ mice (6–8 weeks old; Jackson Laboratory) were infected (titrated frozen stocks with approximately 6.5 log_10_ colony forming units [CFU] of *Mycobacterium tuberculosis* H37Rv) via a burr hole (Micro-Drill Kit; Braintree Scientific, Inc) using a Hamilton syringe (88000; Hamilton) and stereotaxic instrument (model 900, coordinates 0.6 mm dorsal to bregma, 1.2 mm lateral to middle line, and 2 mm ventral; David Kopf Instruments) [5]. Sham mice, uninfected controls to distinguish the effects of the infection and subsequent treatment from those due to the injection procedure itself, received intracranial injections of sterile phosphate-buffered saline (PBS) rather than bacteria, allowing for assessment of baseline inflammatory responses, histopathology, and neuroimaging findings in the absence of infection. All animals were housed in controlled light and temperature rooms without cross-ventilation in a biosafety level-3 facility.

### Antimicrobial Treatments

Drug stocks were prepared and administered 5 days a week (5 consecutive days with 2 days off) via oral gavage. All drugs were purchased from MedChem and were either dissolved or suspended in water. Human equipotent doses of antibitoics were administered via oral gavage in a total volume of 0.2 mL. This dosing in mice produces serum area under the curve (AUC) equivalent to those obtained with currently recommended human doses (Supplementary Table 1) [18, 22, 23]. Dexamethasone was administered intraperitoneally to simulate human equipotent dosing. Bacterial burden was quantified in whole organs as CFU 2 or 6 weeks after treatment initiation using 7H11 plates supplemented with activated charcoal.

### Mass Spectrometry

Tissues were collected at plasma time to maximum concentration (T_max_) for each antibiotic 2 weeks after treatment initiation. Antibiotics were quantified using validated ultra-high–performance liquid chromatography (UPLC) and tandem mass spectrometry (LC-MS/MS) at the Infectious Diseases Pharmacokinetics Laboratory of the University of Florida. The lower limits of detection were 0.25, 0.20, and 0.05 for rifampin, rifapentine, and rifabutin, respectively. Concentrations were normalized to the minimum inhibitory concentration (MICs) for each drug: rifampin at 0.5 μg/mL, rifapentine at 0.125 μg/mL [24], and rifabutin at 0.5 μg/mL [25, 26]. This normalization enables a direct comparison of drug concentrations relative to their antimicrobial potency.

### Dynamic Gadolinium-enhanced Brain MRI

Magnetic resonance imaging (MRI) was used to monitor brain damage and neuroinflammation. Multislice fast spin echo T1-weighted (repetition time [TR] = 1000 ms; echo time [TE] = 11 ms; flip angle = 90°; number of averages = 1; field of view [FOV] = 2 × 2 cm; matrix size = 256 × 248; slice thickness = 1 mm; number of slices = 14–16; fat saturation applied) and fast low-angle shot dynamic contrast-enhanced MRI (TR = 100 ms; TE = 4 ms; flip angle = 25°; number of averages = 1; FOV = 2 × 2 cm; matrix size = 192 × 100; slice thickness = 1 mm; number of slices = 11; time of scan = 20 minutes, intervals of 10 seconds) were performed before and after intravenous injection of gadolinium-based contrast (Magnevist, 0.2 mmol/kg; Bayer) to assess brain changes 2 weeks after treatment initiation. Imaging was performed using a 7T MRI system (MRS DRYMAG; MRSolutions) equipped with a quadrature 20 mm-diameter mouse head coil and custom-built animal biosafety level-3 MRI-compatible biocontainers (Supplementary Figure 1). VivoQuant, Preclinical Scan and Powerscan were used for MRI data analysis.

### Immunofluorescence

Fixed brain tissues collected from animals 2 weeks after treatment initiation were sectioned following overnight incubation at 4°C with primary antibody against Iba-1 (MA5-36257, 1:500; Thermo Fisher). After washing the sections, secondary goat Alexa-fluor 488 antibody (A11034, 1:100; Thermo Fisher) was used to incubate the tissues for 2 hours at room temperature. Sections were washed and mounted with 4′,6-diamidino-2-phenylindole (DAPI) (ProLong Gold Antifade Mountant with DNA Stain DAPI; Thermo Fisher). Zeiss LSM 710 confocal microscope was used at 40× resolution to image. For data analysis, 10 images per sample were processed in FIJI ImageJ to determine the percent area staining for Iba-1.

### Cytokines and Brain Injury Markers

Brain tissue and cerebrospinal fluid (CSF) samples were collected from untreated and treated animals 2 weeks after treatment initiation and stored at −80°C until analysis. Cytokines and chemokines were analyzed using the Meso Scale Discovery plate-based assay on Mesoscale Discovery MESO QuickPlex SQ 120MM Multiplex Reader at the Institute for Clinical and Translational Research, Johns Hopkins University. Brain injury markers (glial fibrillary acidic protein [GFAP] and S100B) were quantified using their respective enzyme-linked immunosorbent assay (ELISA) kits (Thermo Fisher Scientific, EEL098 and EEL109, respectively). Cytokine and chemokine concentrations were quantified using multiplex ELISA targeting a panel of proinflammatory, anti-inflammatory, and tissue injury-associated markers, including interleukin 1β (IL-1β), IL-6, tumor necrosis factor-α (TNF-α), interferon-γ (IFN-γ), IL-2, IL-12, granulocyte-macrophage colony-stimulating factor (GM-CSF), matrix metalloproteinase-9 (MMP-9), monocyte chemoattractant protein-1 (MCP-1), macrophage inflammatory protein-1α (MIP-1α), IL-4, IL-10, and vascular endothelial growth factor (VEGF). Results were normalized to total protein content.

### Statistical Analysis

Data were analyzed using Prism 10.2.2 (GraphPad). Bacterial burden (CFU) is presented on a logarithmic scale (base 10) as mean (SD), and comparisons were performed using a 2-tailed Student *t* test. All other data are presented as median (IQR) and comparisons were performed using a 2-tailed Mann-Whitney *U* test. *P* values ≤ .05 were considered statistically significant.

## RESULTS

### Bacterial Burden

**Figure 1.**
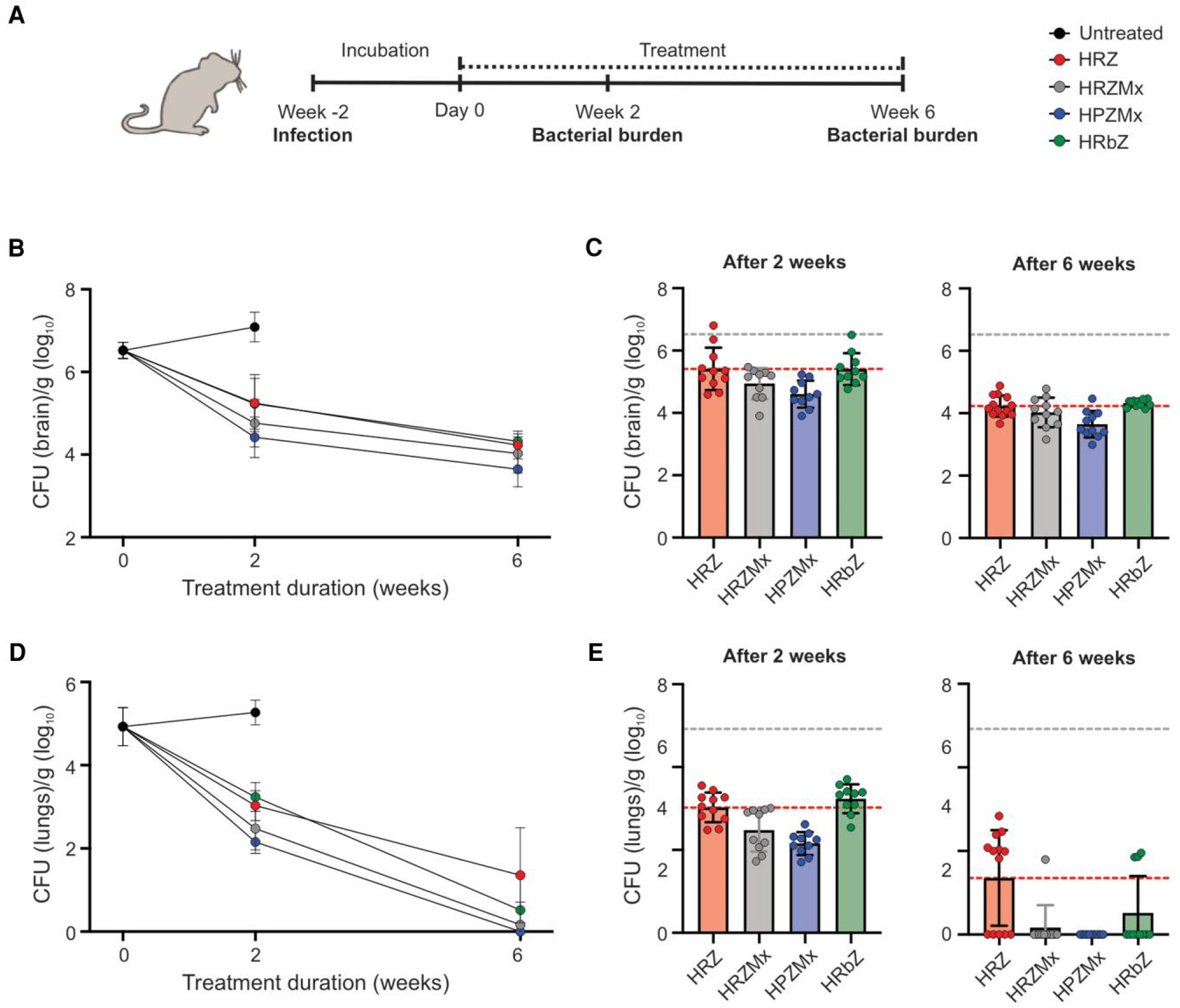
Evaluation of rifamycin-containing regimens. ***A***, Mice were infected intraventricularly via a Burr hole using a syringe and stereotaxic instrument. Treatments were initiated 2 weeks after infection (designated as day 0). Mice were randomly allocated to receive rifamycin-containing regimens, HRZ, HRZMx, HPZMx, or HRbZ, comprising isoniazid (H), pyrazinamide (Z), rifampin (R), rifapentine (P), rifabutin (Rb), and moxifloxacin (Mx) administered orally at human equipotent dosing. All regimens included adjunctive dexamethasone via intraperitoneal injection. At 2 and 6 weeks after treatment initiation, mice were sacrificed, tissues were homogenized and colony forming units (CFU) were enumerated. ***B*** and ***C***, Bacterial burden in brain per gram of tissue (log_10_) from whole brain; animal numbers were n = 11/HRZ, 10/HRZMx, 10/HPZMx, and 10/HRbZ after 2 weeks, and n = 13/HRZ, 11/HRZMx, 11/HPZMx, and 11/HRbZ after 6 weeks. ***D*** and ***E***, Bacteria disseminate to the lungs after brain infection. Therefore, bactericidal activities of the rifamycin-containing regimens in lung tissues of the same animal were also assessed as bacterial burden in lungs per gram of tissue (log_10_) from whole lungs. Data are presented as mean ± SD on a logarithmic scale. *C* and *E*, Each dot represents a single mouse; the bottom dashed line represents the mean values for the standard tuberculosis regimen (HRZ) and the top dashed line indicates the bacterial burden at treatment initiation (day 0).

Human equipotent doses of antibiotics, rifampin [5], rifapentine [18, 27], moxifloxacin [19], rifabutin [20], isoniazid, pyra- zinamide, and dexamethasone [3, 5, 28] were utilized (Supplementary Table 1). Mice with experimentally induced TB meningitis were randomly allocated to receive 1 of the following rifamycin-containing regimen: the standard first-line tuberculosis regimen HRZ; a rifabutin-containing regimen (HRbZ), retaining the conventional HZ backbone; a daily rifapentine-moxifloxacin–containing regimen (HPZMx) approved for pulmonary tuberculosis; or HRZMx, included as a control arm to enable comparative evaluation of the HPZMx regimen (Figure 1A). Dexamethasone was administered with all regimens. Compared to the standard tuberculosis regimen (HRZ), the rifapentine-containing regimen (HPZMx) demonstrated significantly higher bactericidal activity in the brain tissue (*P* = .001), while the activity of the rifabutin-containing regimen (HRbZ) was similar to HRZ (*P* = .398) (Figure 1B and 1C, and Supplementary Tables 2 and 3). The addition of moxifloxacin (HRZMx) to the standard tuberculosis regimen (HRZ) did not improve the activity of the regimen in the brain tissue (*P* = .236).

Because bacteria disseminate to the lungs after a brain infection [5, 17], we compared the bactericidal activity of each regimen in the lungs of the same animals. The activity of all 3 regimens was better in the lung compared to the brain tissues (Figure 1D and 1E). Compared to the standard tuberculosis regimen (HRZ), the rifapentine-containing regimen (HPZMx) demonstrated significantly higher bactericidal activity in the lung tissue (*P* ≤ .001), while the activity of the rifabutin-containing regimen (HRbZ) was similar (*P* = .060). Addition of moxifloxacin (HRZMx) to the standard tuberculosis regimen (HRZ) significantly improved the activity of the regimen in the lung tissue (*P* = .004) (Figure 1E), an effect that was not observed in the brain tissues.

### Tissue Antibiotic Concentrations

**Figure 2.**
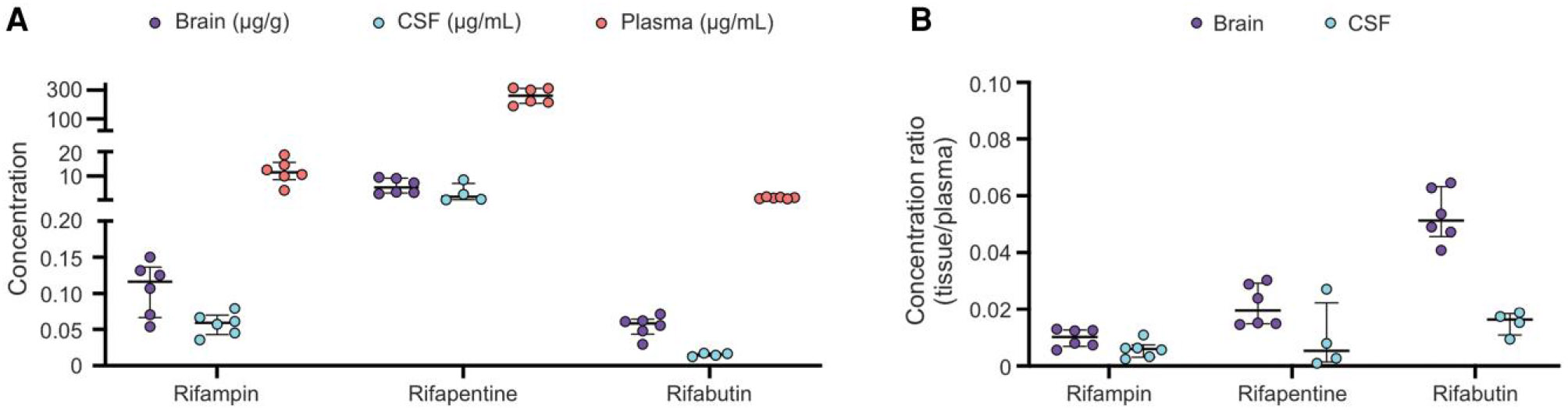
Rifamycin tissue concentrations. ***A***, Brain, cerebrospinal fluid (CSF), and plasma concentrations measured by mass spectrometry in the mouse model of tuberculosis meningitis 2 weeks after treatment initiation, normalized to the corresponding minimum inhibitory concentration for each drug (rifampin, 0.5 μg/mL; rifapentine, 0.125 μg/mL; and rifabutin, 0.5 μg/mL); animal numbers were n = 6 animals/regimen for brain and plasma tissues; n = 6/rifampin, 4/rifapentine, and 4/rifabutin for CSF). ***B***, Tissue-to-plasma ratios from brain and CSF for each rifamycin. Data are presented as median ± IQR. Each dot represents a single mouse.

The brain and CSF concentrations of all rifamycins were lower than in plasma, suggesting restricted CNS penetration (Figure 2A). All 3 rifamycins achieved higher concentrations in brain tissue versus the CSF (Figure 2B). In the lungs, all rifamycins achieved markedly higher concentrations compared to brain, with rifapentine levels much higher compared to rifampin and rifabutin (Supplementary Figure 2). Given substantial differences in the MIC for the 3 rifamycins, we normalized all 3 drugs to their corresponding MIC (rifampin, 0.5 μg/mL; rifapentine, 0.125 μg/mL; and rifabutin: 0.5 μg/mL).

### Dynamic Gadolinium-enhanced Brain MRI

**Figure 3.**
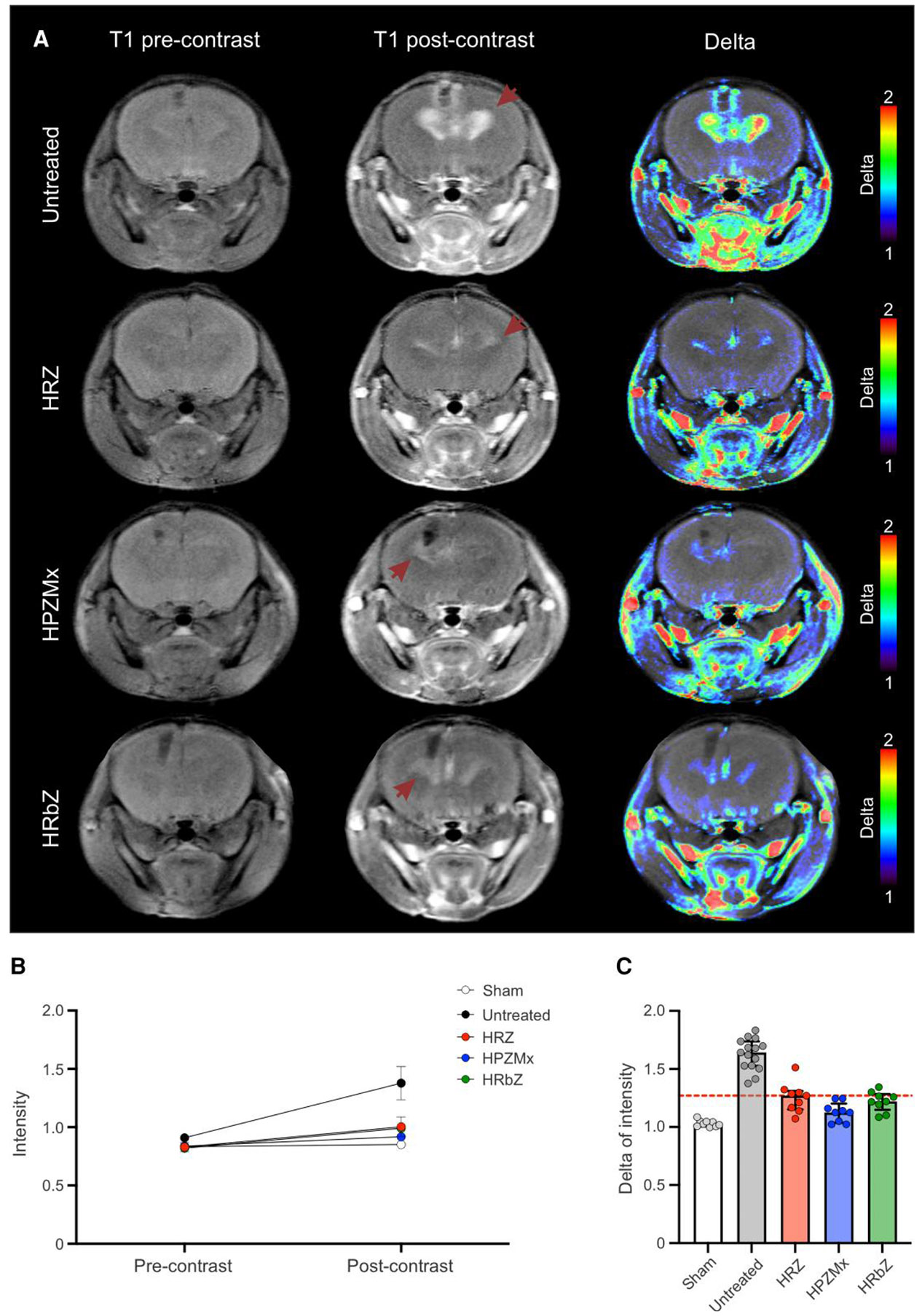
Dynamic gadolinium-enhanced brain magnetic resonance imaging (MRI). Mice were randomly allocated to receive rifamycin-containing regimens, HRZ, HPZMx, or HRbZ. MRI was performed 2 weeks after treatment initiation. Data from infected but untreated, and uninfected (phosphate-buffered saline injected, sham) animals are also shown (animal numbers were n = 3/sham, 5/untreated, 3/HRZ, 3/HPZMx, and 3/HRbZ; 3 volumes of interest per animal). ***A***, Representative axial T1-weighted MRI acquired precontrast (left column) and postcontrast (middle column) with corresponding delta maps (right column) demonstrating contrast enhancement at the infection sites (periventricular) in the brains of infected animals. Arrows indicate areas of periventricular contrast enhancement. ***B***, Quantitative analysis of mean signal intensity normalized by brain background before and after contrast administration across groups. ***C***, Delta of intensity (postcontrast divided by precontrast) for each group. Data are presented as median ± IQR. The dashed line represents the median values for the standard tuberculosis regimen (HRZ). Abbreviations: HPZMx, isoniazid, rifapentine, pyrazinamide, moxifloxacin; HRbZ, isoniazid, rifabutin, pyrazinamide; HRZ, isoniazid, rifampin, pyrazinamide.

We performed dynamic gadolinium-enhanced brain MRI in live mice undergoing tuberculosis treatments due to its clinical relevance in detecting neuroinflammation and BBB disruption [29-31]. As expected, infected but untreated animals demonstrated a marked increase in contrast enhancement at the site of infection (periventricular region) (Supplementary Figure 3A), which was evident as early as 1 minute after contrast injection and persisted throughout the duration of the scan. Conversely, uninfected animals (sham controls injected with PBS) exhibited minimal changes. Quantitative analysis confirmed a significant rise in signal intensity over time in the periventricular brain region of infected animals versus uninfected animals (sham controls) (*P* < .001). Stable signal intensities were also observed in control tissues (uninfected brain and muscle) in the infected animals (Supplementary Figure 3B). Postcontrast and delta maps (precontrast images subtracted from postcontrast images) further highlighted this localized brain enhancement in infected animals (Supplementary Figure 3C). All treatment regimens reduced contrast enhancement in the infected animals, with the most significant effect observed in the rifapentine-containing regimen (*P* = .018), suggesting reduced inflammation (Figure 3).

### Inflammatory Markers

**Figure 4.**
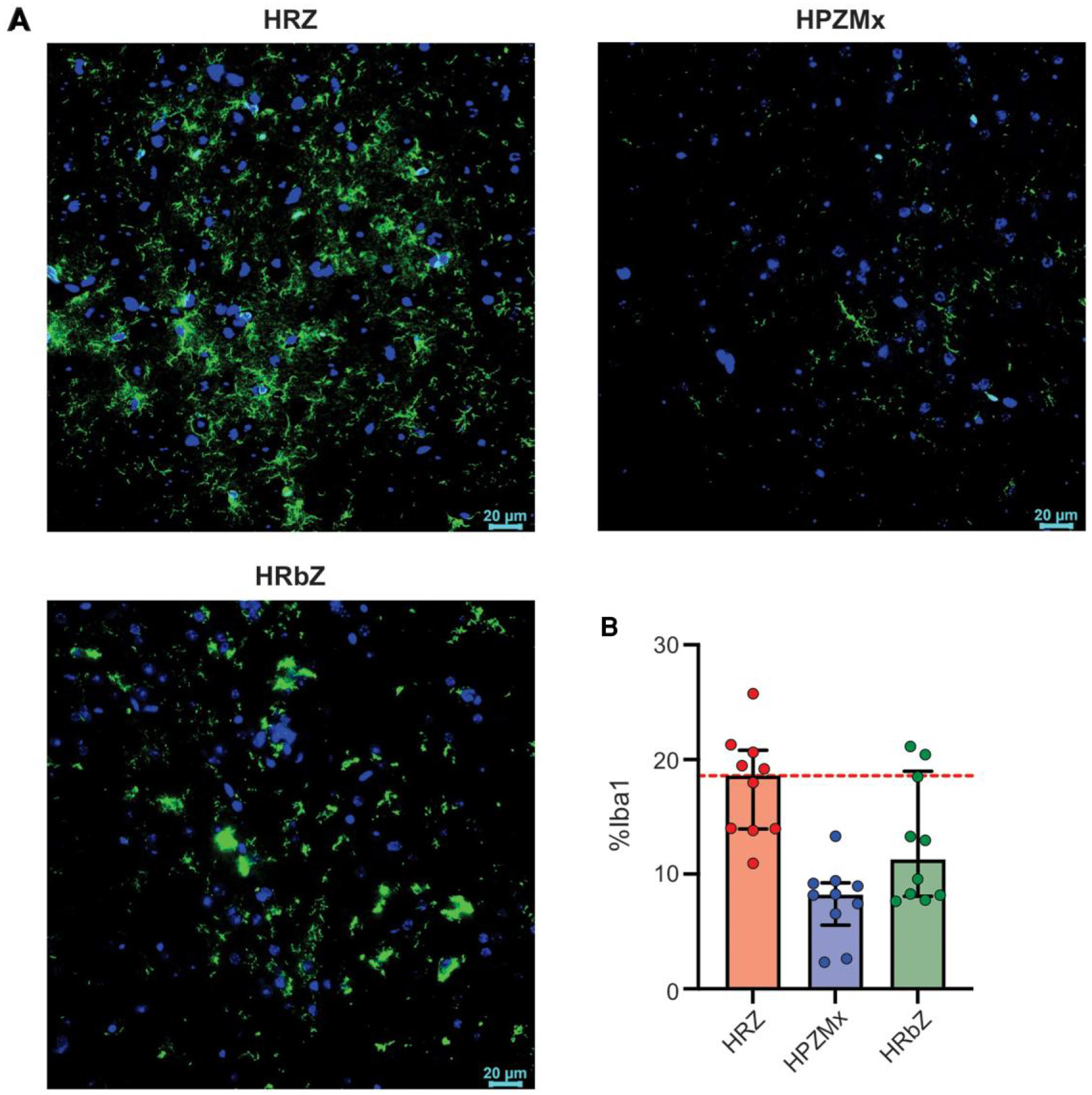
Microglial activation in brain tissues. Mice were randomly allocated to receive rifamycin-containing regimens, HRZ, HPZMx, or HRbZ. Brain tissues obtained 2 weeks after treatment initiation were stained (10 sections per group). ***A***, Representative immunofluorescence images and quantification of Iba-1 in the mouse model of tuberculous meningitis. Microglia were labeled with Iba-1 (green), and nuclei were counterstained with DAPI (blue). ***B***, Quantification (% Iba-1 area) for each group. Data are presented as median ± IQR. The dashed line represents the median values for the standard tuberculosis regimen (HRZ). Abbreviations: HPZMx, isoniazid, rifapentine, pyrazinamide, moxifloxacin; HRbZ, isoniazid, rifabutin, pyrazinamide; HRZ, isoniazid, rifampin, pyrazinamide.

Immunofluorescence in postmortem brain tissues demonstrated that Iba-1 staining was reduced in animals treated with all the rifampin-containing regimens. However, brains tissues from animals treated with either rifapentine (HPZMx)- or rifabutin (HRbZ)-containing regimens exhibited visibly reduced Iba-1 immunoreactivity (Figure 4). Quantitative analysis confirmed that Iba-1 staining was significantly lower in brain tissues from animals treated with the rifapentine- containing (HPZMx; *P* < .001) or rifabutin-containing (HRbZ; *P* = .036) regimens compared to the standard tuberculosis regimen (HRZ).

**Figure 5.**
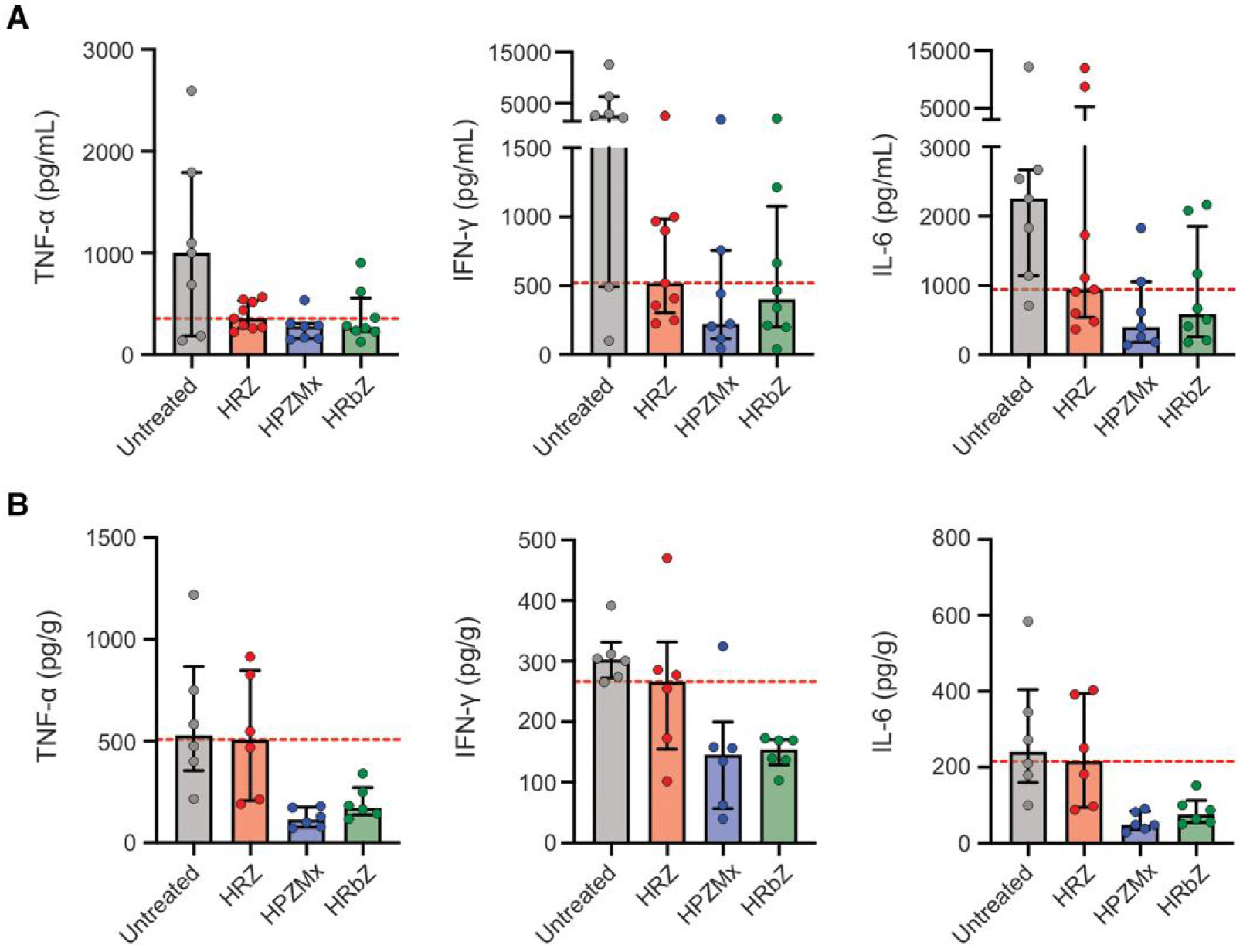
Brain and CSF tissue inflammatory markers. Mice were randomly allocated to receive rifamycin-containing regimens, HRZ, HPZMx, or HRbZ. Brain tissue and CSF were obtained 2 weeks after treatment initiation. Data from infected but untreated animals are also shown. ***A***, Proinflammatory cytokines in CSF (animal numbers were n = 7/untreated, 9/HRZ, 7/HPZMx, and 8/HRbZ). ***B***, Proinflammatory cytokines in brain tissues (animal numbers were n = 6 animal/regimen). Data are presented as median ± IQR. Each dot represents a single mouse. The dashed line represents the median values for the standard tuberculosis regimen (HRZ). Abbreviations: CSF, cerebrospinal fluid; HPZMx, isoniazid, rifapentine, pyrazinamide, moxifloxacin; HRbZ, isoniazid, rifabutin, pyrazinamide; HRZ, isoniazid, rifampin, pyrazinamide; IL, interleukin; TNF, tumor necrosis factor; IFN, interferon.

Analysis of cytokine and chemokine profiles in the CSF and brain revealed marked differences between untreated and treated groups. Untreated animals exhibited high levels of proinflammatory cytokines, including TNF-α, IFN-γ, and IL-6 (Figure 5A and 5B). All treatment regimens reduced these cytokines levels to varying degrees in both CSF and brain tissues, with the rifapentine-containing (HPZMx) or rifabutin-containing (HRbZ) regimens generally showing lower median values than the standard tuberculosis regimen (HRZ) (Supplementary Table 4). Both HPZMx and HRbZ regimens led to notable reductions in TNF-α, while HPZMx also appeared particularly effective at lowering IFN-γ and IL-6. Other proinflammatory cytokine (IL-1β), Th1/Th17-associated cytokines (IL-2, IL-12, GM-CSF), and MMP-9 followed similar trends, with the alternative rifamycin regimens (HPZMx and HRbZ) achieving the greatest suppression (Supplementary Figures 4A and 5A). Chemokines such as MCP-1 and MIP-1α were also reduced in all treated groups, with HPZMx showing the most pronounced effect (Supplementary Figures 4B and 5B). Similarly, VEGF levels were lowest in the HRbZ group (Supplementary Figures 4C and 5C) [20], and, in contrast, anti-inflammatory cytokines (IL-4 and IL-10) did not differ significantly between groups (Supplementary Figures 4D and 5D).

### Neuronal Injury Markers

**Figure 6.**
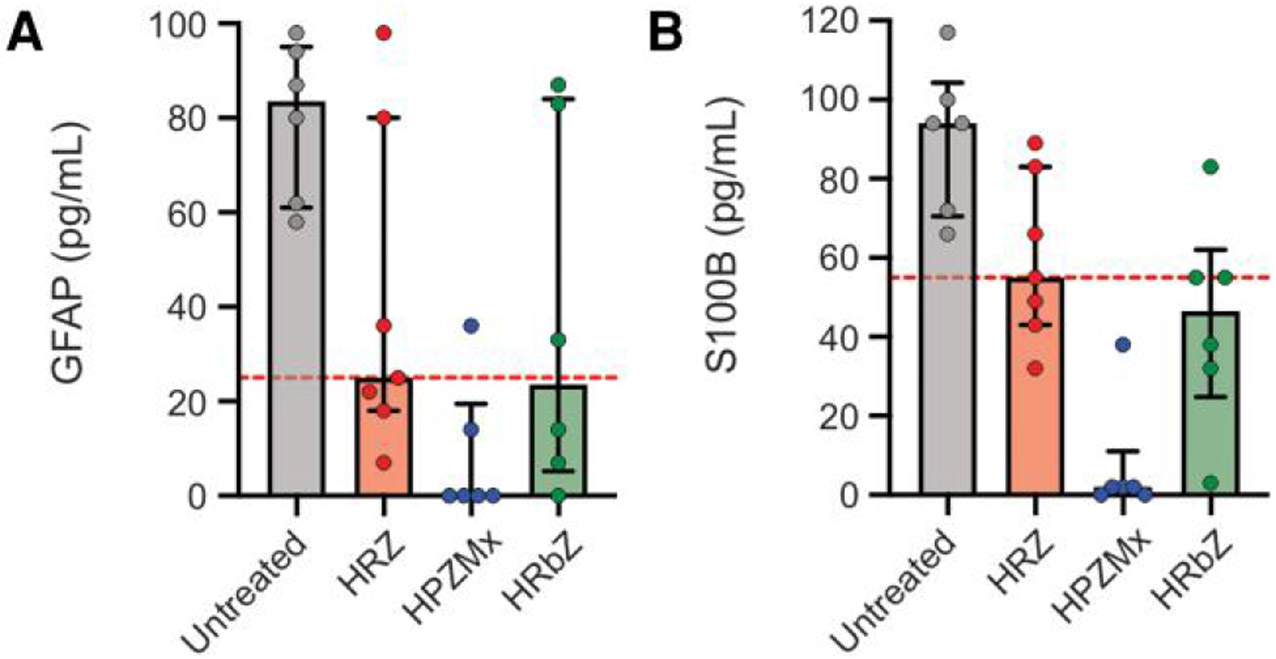
Plasma neuronal injury markers. Mice were randomly allocated to receive rifamycin-containing regimens, HRZ, HPZMx, or HRbZ. Brain tissues were obtained 2 weeks after treatment initiation. Data from infected but untreated animals are also shown. *A*, GFAP and (*B*) S100B levels in plasma (animal numbers were n = 6/untreated, 7/HRZ, 6/HPZMx, and 6/HRbZ). Data are presented as median ± IQR. Each dot represents a single mouse. The dashed line represents the median values for the standard tuberculosis regimen (HRZ). Abbreviations: GFAP, glial fibrillary acidic protein; HPZMx, isoniazid, rifapentine, pyrazinamide, moxifloxacin; HRbZ, isoniazid, rifabutin, pyrazinamide; HRZ, isoniazid, rifampin, pyrazinamide.

Serum GFAP and S100B were included as clinically relevant blood biomarkers of CNS injury [32, 33]. While untreated animals had high serum levels of neuronal injury markers GFAP and S100B, all treatment regimens reduced these markers to varying degrees, with the rifapentine-containing (HPZMx) regimen being most effective, resulting in the lowest and least variable levels of both GFAP and S100B (Figure 6 and Supplementary Table 4).

## DISCUSSION

Although increasing rifampin doses has shown to enhance bactericidal activity in mouse and rabbit models [5, 17], as well as improve mortality or neurological outcomes in phase 2 clinical trials in adults and children, respectively [7, 10], these benefits were not translated into improved clinical outcomes (mortality or neurological deficits) in a recent, larger clinical trial [12]. Although the exact reason(s) for the lack of benefit from high-dose rifampin in this recent clinical trial are unknown, it is hypothesized that reduced corticosteroids exposure in patients with TB meningitis, resulting from rifampin-induced hepatic clearance, may be responsible. In this context, rifabutin may be beneficial, due to its weaker induction of hepatic metabolism relative to rifampin [34-37], and potentially enabling greater corticosteroid exposure in patients with TB meningitis. The present study demonstrates that alternative rifamycin-containing regimens, particularly those incorporating rifapentine or rifabutin, can match the early bactericidal activity of the standard rifampin-based tuberculosis regimen (HRZ) for TB meningitis, while also favorably modulating neuroinflammation and brain injury.

Rifabutin was directly compared with the standard rifampin-containing regimen (HRZ) by substituting the rifamycin (HRbZ). In contrast, rifapentine was evaluated in the clinically approved pulmonary tuberculosis regimen combined with moxifloxacin (HPZMx) [16], using a comparable rifampin-containing regimen (HRZMx) as the control. Overall, 2 main conclusions emerge. First, substituting rifabutin for rifampin within an HZ backbone provides similar bacterial killing in the brain. Second, although neither the addition of moxifloxacin to the standard rifampin-containing regimen (HRZ) nor the substitution of rifapentine for rifampin in the HRZMx regimen on its own significantly improve brain antibacterial activity, the clinically approved rifapentine-moxifloxacin–containing regimen (HPZMx) was associated with a lower brain bacterial burden compared to the HRZ regimen, suggesting additive contributions from both moxifloxacin and rifapentine. This is consistent with clinical trial data from rifapentine-containing regimens for pulmonary tuberculosis, where substitution of rifampin with rifapentine failed to demonstrate noninferiority whereas the rifapentine-moxifloxacin–containing regimen (HPZMx) arm did show benefit over the standard tuberculosis treatment (HRZ) [16].

Rifampin, rifapentine, and rifabutin share the rifamycin naphthalene *ansa* core and inhibit the β-subunit of bacterial DNA-dependent RNA polymerase [38, 39], but have distinct C3-C4 side chains conferring differences in lipophilicity, protein binding, biodistribution, and half-life. Rifampin is lipophilic with substantial plasma protein binding, rifapentine is even more lipophilic with higher protein binding and prolonged systemic exposure, while rifabutin combines high lipophilicity with comparatively lower protein binding. These properties also influence unbound tissue drug availability and may contribute to the observed differences in brain tissue versus CSF exposure among the 3 drugs. Consistent with prior data on CNS exposures of tuberculosis drugs [3, 5, 6, 15], all rifamycins achieved higher brain tissue levels than in the CSF, underscoring again that CSF alone may not correctly estimate true CNS exposure for protein-bound, lipophilic drugs [5, 6, 15]. These findings align with clinical data demonstrating substantial within-CNS variability of rifampin concentrations across lumbar CSF, ventricular CSF, and cerebral extracellular fluid [40, 41], highlighting the limitations of relying solely on lumbar CSF as a surrogate for parenchymal exposure [42]. Furthermore, the activities of these regimens remained greater in the lungs than in the brain, underscoring the ongoing challenge of developing CNS-specific regimens for tuberculosis [1, 43].

This study has some limitations. Only total rifamycin concentrations were measured in plasma, lung, brain tissue, and CSF. However, only the unbound drug is pharmacologically active and can cross the blood-brain and blood-CSF barriers. Quantifying the very low unbound fractions of highly protein-bound rifamycins remains analytically challenging, so the CNS pharmacokinetic/pharmacodynamic inferences here are based on total rather than unbound concentrations. Furthermore, tissue-to-plasma ratio, where the unbound (but presumably not the protein-bound) fraction crosses, were calculated to enable relative comparisons among rifampin, rifapentine, and rifabutin CNS exposures. We utilized the 5-day-on/2-day-off dosing schedule for administrating antibiotics treatments, which is commonly used in mouse models of tuberculosis. However, this may not fully mimic an uninterrupted 7-day daily dosing. The longer half-life of rifapentine, in particular, may influence drug exposure during nondosing intervals and could have impacted outcomes. Although bactericidal activity and inflammatory parameters were assessed, this study did not directly evaluate survival or neurological function. Nonetheless, gadolinium-enhanced contrast MRI, a sensitive and clinically relevant marker of neuroinflammation [29-31], may serve as an adjunctive biomarker in future clinical trials evaluating new therapeutics for TB meningitis.

In summary, our findings support that, within clinically relevant backbones, alternative rifamycins, particularly rifabutin and rifapentine, may offer important advantages over standard rifampin-based treatment for TB meningitis. These findings provide strong rationale for further clinical evaluation of rifapentine- and rifabutin-based regimens using integrated bacteriological, imaging, and clinical end points to guide rifamycin selection and dosing.

## Supplementary Material

Supplementary Data

Supplementary materials are available at The Journal of Infectious Diseases online (http://jid.oxfordjournals.org/). Supplementary materials consist of data provided by the author that are published to benefit the reader. The posted materials are not copyedited. The contents of all supplementary data are the sole responsibility of the authors. Questions or messages regarding errors should be addressed to the author.

## Data Availability

All data needed to evaluate the conclusions in the paper are present in the paper and/or the Supplementary Materials.
