# Supplementary material for: Beyond Rifampin: Evaluating Rifapentine and Rifabutin as Alternative Treatments for Tuberculous Meningitis
Source: J Infect Dis. 2026 Feb 10;234(1):e170–9. doi: 10.1093/infdis/jiag087 (PMC13086524; doi:10.1093/infdis/jiag087)
Supplement: jiag087_Supplementary_Data [file jiag087_supplementary_data.docx]

Supplementary Figures

**
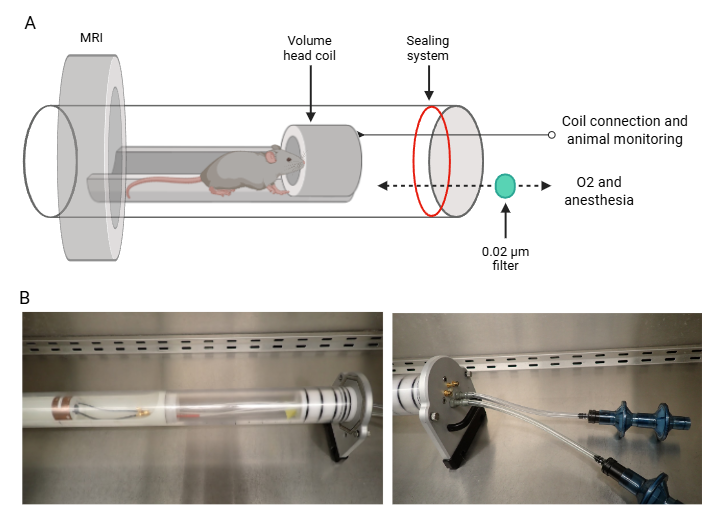
**

**Figure S1. Custom-built MRI-compatible biocontainer.** (**A**) Schematic of the custom-built biosafety level-3 (BSL-3) MRI-compatible biocontainer. The biocontainer is sealed (air-tight) for BSL-3 containment and has filters (0.02 µm) at the inlet and the outlet allowing the delivery of air-anesthesia mix. (**B**) Actual bed with a head volume coil for mouse imaging. The workflow ensures animal and personnel safety during the imaging studies.


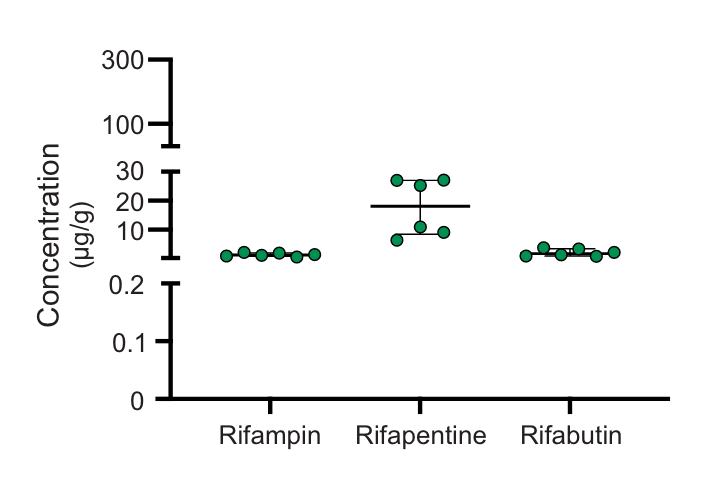


**Figure S2. Rifamycin lung concentrations.** **Rifamycin lung concentrations.** Lung concentrations measured by mass spectrometry in *M. tuberculosis*-infected mice with TB meningitis two weeks after treatment initiation, normalized to the corresponding minimum inhibitory concentration (MIC) for each drug (rifampin: 0.5 μg/mL, rifapentine: 0.125 μg/mL, and rifabutin: 0.5 μg/mL) (animal number, *n* =  6 animals/regimen) Data are represented as median ± interquartile range. Each dot represents a single mouse.


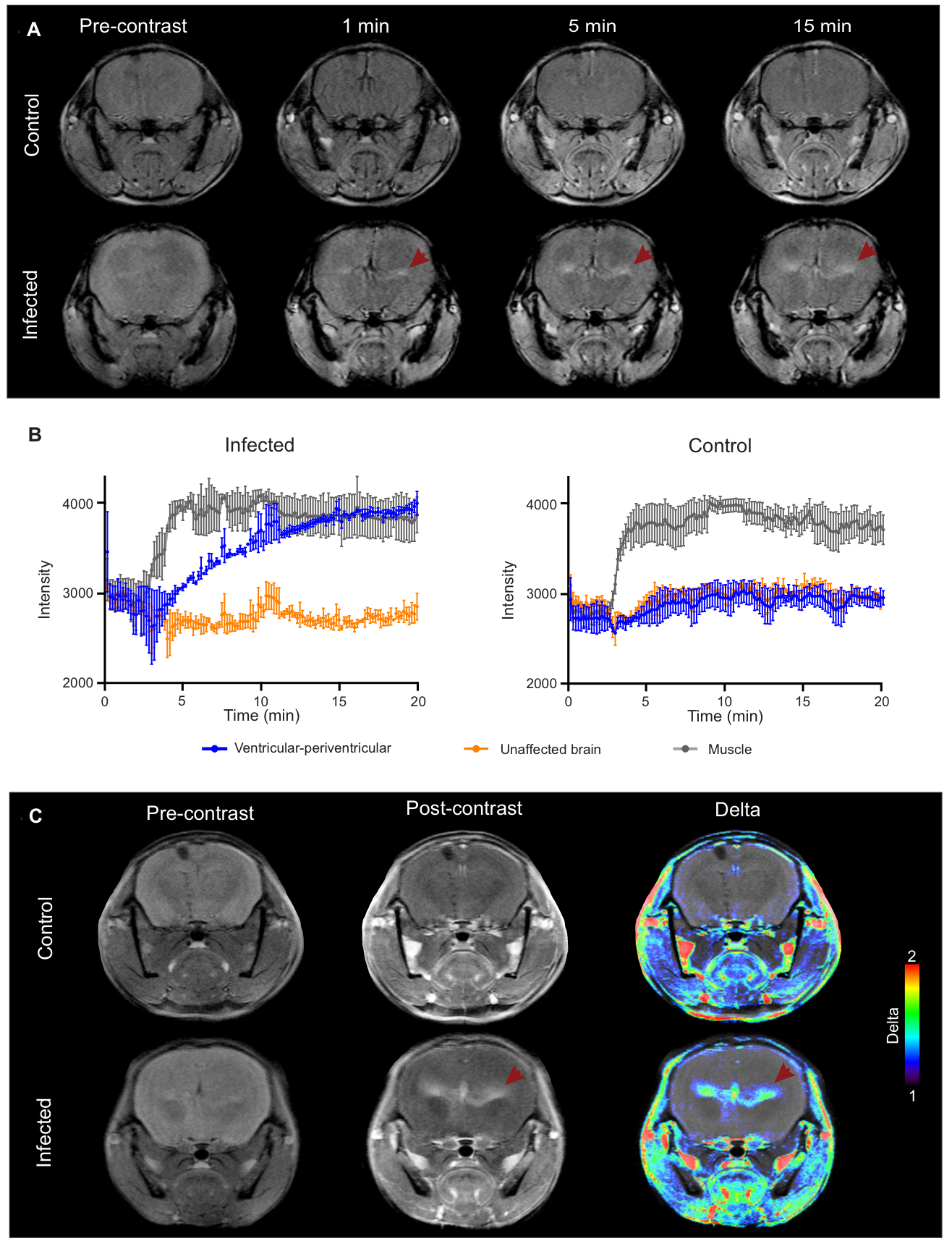


Figure S3. Dynamic gadolinium-enhanced brain MRI. (A) Representative axial MRI images from uninfected (PBS injected, “sham”) and infected animals at pre-contrast, 1-, 5- and 15-min post-contrast administration. Red arrowheads indicate regions of increased brain signal intensity in the periventricular area (site of infection) in infected animals. (B) Quantification of signal intensity over time in periventricular area, unaffected brain, and muscle. (C) Axial MRI images showing pre-contrast, post-contrast, and delta (change in signal intensity normalized by brain background) for control and infected animals. The delta highlights areas of increased contrast uptake at the infection site (red arrowhead).


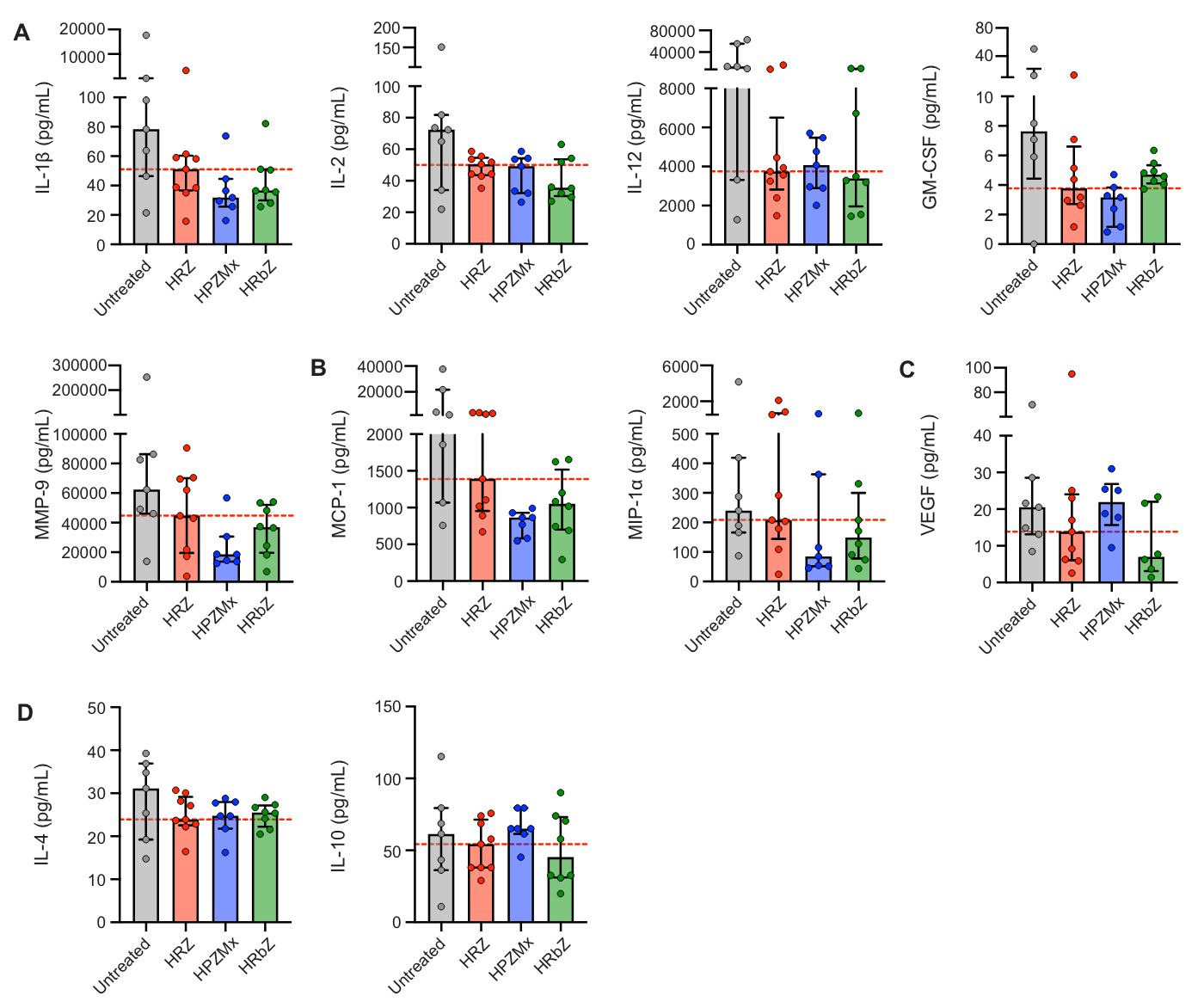
Figure S4. CSF inflammatory markers. Mice were randomly allocated to receive rifamycin-containing regimens, HRZ, HPZMx, or HRbZ. CSF was obtained two weeks after treatment initiation. Data from infected, but untreated animals are also shown. (A) Pro-inflammatory cytokines, (B) Chemokines, (C) Vascular endothelial growth factor (VEGF), and (D) Anti-inflammatory cytokines. Six animals per regimen were utilized. Data are presented as median ± interquartile range. Each dot represents a single mouse. The red dashed line represents the median values for the standard TB regimen (HRZ).


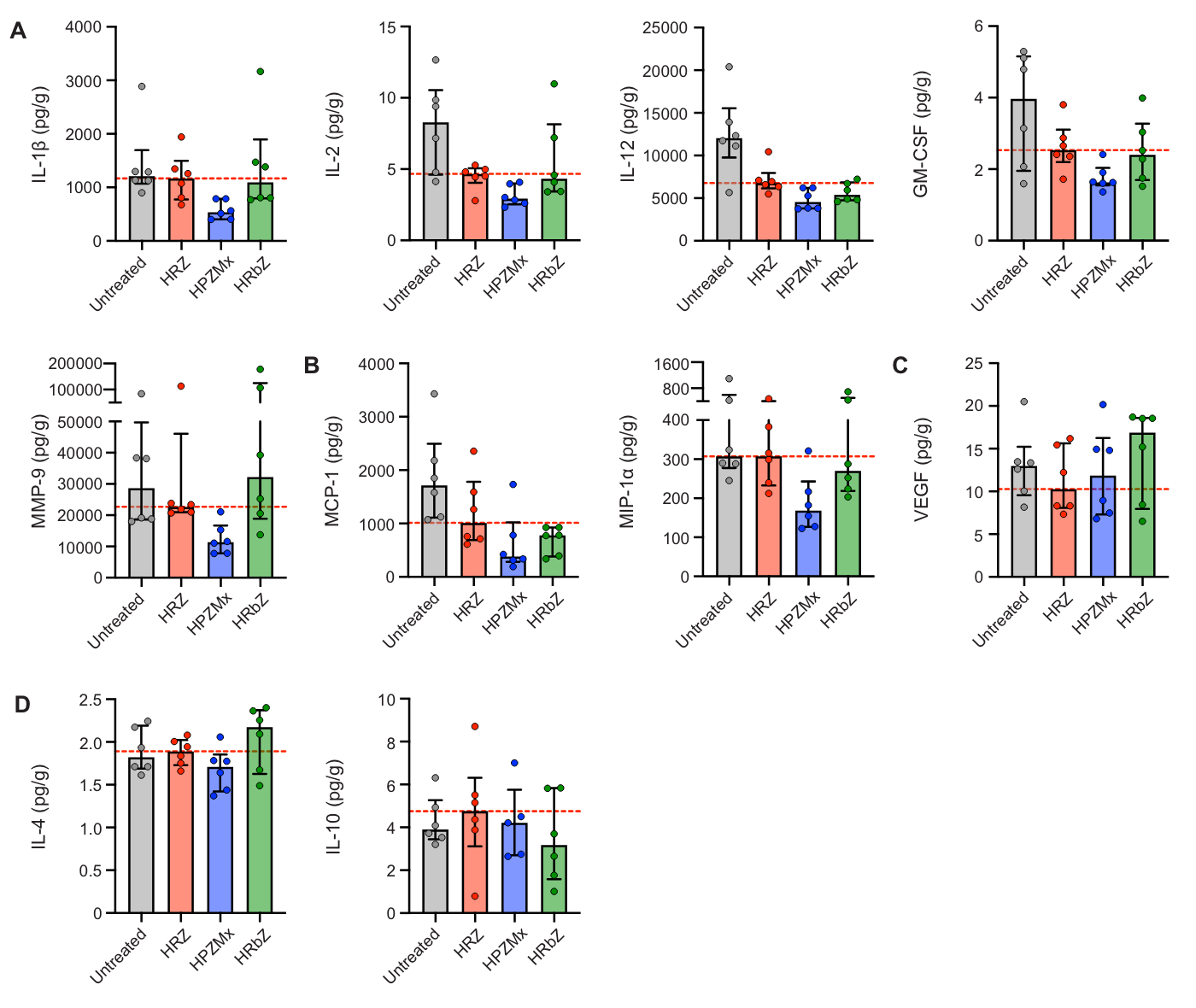
Figure S5. Brain tissue inflammatory markers. Mice were randomly allocated to receive rifamycin-containing regimens, HRZ, HPZMx, or HRbZ. Brain tissues were obtained two weeks after treatment initiation. Data from infected, but untreated animals are also shown. (A) Pro-inflammatory cytokines, (B) Chemokines, (C) Vascular endothelial growth factor (VEGF), and (D) Anti-inflammatory cytokines. Seven, nine, seven and eight animals were utilized for the untreated, HRZ, HPZMx and HRbZ groups, respectively. Data are presented as median ± interquartile range. Each dot represents a single mouse. The red dashed line represents the median values for the standard TB regimen (control group, HRZ).

**Supplementary Tables**

**Table S1. Antibiotic dosing.** Doses used in mice with the corresponding human equipotent doses. BID = twice a day.

| **Table S1. Antibiotic dosing** | | |
| --- | --- | --- |
| **Drug** | **Mouse dose** | **Human dose** |
| Isoniazid | 10 mg/kg/day | 10 mg/kg |
| Pyrazinamide | 150 mg/kg/day | 25 mg/kg |
| Rifampin | 10 mg/kg/day | 10 mg/kg |
| Rifapentine | 20 mg/kg/day | 1200 mg |
| Rifabutin | 10 mg/kg/day | 300 mg |
| Moxifloxacin | 100 mg/kg/day divided BID | 400 mg |
| Dexamethasone | 2 mg/kg/day | 0.4 mg/kg |

**Table S2. Bacterial burden at two weeks of treatment in mice.** Colony-forming unit (CFU) per gram of tissue (log_10_) from whole brain and lung tissues two weeks after treatment initiation. The bacterial burden at the treatment initiation for brain and lung tissues was 6.521 ± 0.129 and 4.927 ± 0.458 log_10_ CFU/gm, respectively. HRZ (isoniazid, rifampin, pyrazinamide), HPZMx (isoniazid, rifapentine, pyrazinamide, moxifloxacin), and HRbZ (isoniazid, rifabutin, pyrazinamide). Statistical comparisons were performed using a two-tailed student t test.

| **Table S2. Bacterial burden at two weeks of treatment in mice** | | | | | | |
| --- | --- | --- | --- | --- | --- | --- |
| **Group** | **Mean** | **Standard deviation** | **Group** | **Mean** | **Standard deviation** | **Adjusted**  ***P* value** |
| **Brain** | | | | | | |
| **HRZ** | 5.41 | 0.68 | **HRZMx** | 4.94 | 0.50 | 0.090 |
|  |  |  | **HPZMx** | 4.60 | 0.43 | 0.004 |
|  |  |  | **HRbZ** | 5.41 | 0.51 | 0.997 |
| **HRZMx** | 4.94 | 0.50 | **HPZMx** | 4.60 | 0.43 | 0.122 |
| **Lungs** | | | | | | |
| **HRZ** | 3.03 | 0.36 | **HRZMx** | 2.48 | 0.51 | 0.010 |
|  |  |  | **HPZMx** | 2.16 | 0.28 | <0.001 |
|  |  |  | **HRbZ** | 3.23 | 0.35 | 0.197 |
| **HRZMx** | 2.48 | 0.51 | **HPZMx** | 2.16 | 0.28 | 0.098 |

**Table S3. Bacterial burden at six weeks of treatment in mice.** Colony-forming unit (CFU) per gram of tissue (log_10_) from whole brain and lung tissues six weeks after treatment initiation. The bacterial burden at the treatment initiation for brain and lung tissues was 6.521 ± 0.129 and 4.927 ± 0.458 log_10_ CFU/gm, respectively. HRZ (isoniazid, rifampin, pyrazinamide), HPZMx (isoniazid, rifapentine, pyrazinamide, moxifloxacin), and HRbZ (isoniazid, rifabutin, pyrazinamide). Statistical comparisons were performed using a two-tailed student t test.

| **Table S3. Bacterial burden at six weeks of treatment in mice** | | | | | | |
| --- | --- | --- | --- | --- | --- | --- |
| **Group** | **Mean** | **Standard deviation** | **Group** | **Mean** | **Standard deviation** | **Adjusted**  ***P* value** |
| **Brain** | | | | | | |
| **HRZ** | 4.23 | 0.34 | **HRZMx** | 4.03 | 0.47 | 0.236 |
|  |  |  | **HPZMx** | 3.65 | 0.43 | 0.001 |
|  |  |  | **HRbZ** | 4.32 | 0.11 | 0.398 |
| **HRZMx** | 4.03 | 0.47 | **HPZMx** | 3.65 | 0.43 | 0.061 |
| **Lungs** | | | | | | |
| **HRZ** | 1.35 | 1.14 | **HRZMx** | 0.16 | 0.54 | 0.004 |
|  |  |  | **HPZMx** | 0.00 | 0.00 | <0.001 |
|  |  |  | **HRbZ** | 0.52 | 0.88 | 0.060 |
| **HRZMx** | 0.16 | 0.54 | **HPZMx** | 0.00 | 0.00 | 0.329 |

**Table S4. Biomarker comparisons.** Comparison for biomarkers measured in the brain [μg/g; median ± interquartile range (IQR)], and serum (μg/mL; median ± IQR) calculated for HRbZ (isoniazid, rifabutin, pyrazinamide) or HPZMx (isoniazid, rifapentine, pyrazinamide, moxifloxacin) treatment groups compared with standard HRZ (isoniazid, rifampin, pyrazinamide) treatment in the mouse model of TB meningitis. Statistical comparisons were performed using a two-tailed Mann-Whitney U test.

| **Table S4. Tissue inflammatory markers** | | | | | | | | | |
| --- | --- | --- | --- | --- | --- | --- | --- | --- | --- |
| **Tissue** | **Marker** | **HRZ** | | **HRbZ** | | | **HPZMx** | | |
|  |  | **Median** | **IQR** | **Median** | **IQR** | ***P* value** | **Median** | **IQR** | ***P* value** |
| **Brain** | **TNFα** | 506.7 | 479.1 | 172.7 | 83.6 | 0.026 | 113.9 | 78.5 | 0.002 |
|  | **IFNγ** | 266 | 90.5 | 154.2 | 31.6 | 0.093 | 145.6 | 77 | 0.132 |
|  | **IL-6** | 215.5 | 238.3 | 74.9 | 39.1 | 0.026 | 48.4 | 33.4 | 0.004 |
|  | **IL-1β** | 1166.8 | 449.7 | 1095.7 | 645.7 | 0.181 | 537.8 | 299.7 | 0.009 |
|  | **IL-2** | 4.7 | 0.5 | 4.3 | 2.9 | >0.999 | 2.9 | 1.1 | 0.026 |
|  | **IL-12** | 6764.2 | 649.4 | 5410.4 | 1697.2 | 0.179 | 4561.4 | 2154 | 0.009 |
|  | **GM-CSF** | 2.5 | 0.4 | 2.4 | 1 | 0.818 | 1.6 | 0.2 | 0.017 |
|  | **MMP-9** | 22671.8 | 2471.8 | 32253.8 | 68465 | 0.699 | 11342.9 | 5732.9 | 0.009 |
|  | **MCP-1** | 1010.9 | 784.9 | 777.5 | 398.8 | 0.393 | 379.8 | 372.6 | 0.132 |
|  | **MIP-1α** | 307.1 | 111.6 | 270 | 169 | 0.818 | 168.4 | 73.7 | 0.041 |
|  | **VEGF** | 10.3 | 6.3 | 16.9 | 8.4 | 0.309 | 11.9 | 7 | >0.999 |
|  | **IL-4** | 1.9 | 0.2 | 2.2 | 0.6 | 0.309 | 1.7 | 0.3 | 0.179 |
|  | **IL-10** | 4.8 | 1.4 | 3.2 | 3.3 | 0.589 | 4.2 | 1.8 | 0.662 |
| **Serum** | **GFAP** | 25 | 38 | 23.5 | 61.8 | 0.657 | 0 | 10.5 | 0.025 |
|  | **S100B** | 55 | 28.5 | 46.5 | 21.5 | 0.344 | 2 | 1.5 | 0.002 |
